# Treatment-Induced Gene Expression Changes in Metastatic Renal Cell Carcinoma: Insights from a Syngeneic Mouse Model

**DOI:** 10.3390/curroncol32070391

**Published:** 2025-07-08

**Authors:** Ko Okabe, Toshiaki Tanaka, Tetsuya Shindo, Yuki Kyoda, Sachiyo Nishida, Kohei Hashimoto, Ko Kobayashi, Naoya Masumori

**Affiliations:** Department of Urology, Sapporo Medical University, South-1, West-16, Chuo-ku, Sapporo 060-8543, Japan; kookabe0408@yahoo.co.jp (K.O.);

**Keywords:** gene expression profiles, immune checkpoint inhibitor, metastatic renal cell carcinoma, mouse model, targeted therapy

## Abstract

This study aimed to clarify the alterations in gene expression in metastatic renal cell carcinoma (mRCC) during disease progression and in response to treatment with immune checkpoint inhibitors using a syngeneic mouse mRCC model. RENCA cells were orthotopically implanted in BALB/c mice. Mice received first-line treatment with cabozantinib, anti-PD-1 antibody, or a combination. Tumor progression was monitored using serial micro-computed tomography. Lung metastasis samples were collected, and RNA sequencing was performed. Mice with apparent disease progression received second-line treatment with axitinib, everolimus, or lenvatinib after combination therapy. The median overall survival was 28, 34, 34, and 49 days in untreated mice and those treated with cabozantinib, anti-PD-1, or their combination, respectively (*p* < 0.05). RNA sequencing revealed upregulation of the fibroblast growth factor pathway in lung metastases after monotherapy, whereas mTOR pathway activation was observed only after combination therapy. Treatment-specific gene expression changes occur in mRCC, suggesting that the optimal target for sequential therapy in mRCC varies depending on prior treatment.

## Simple Summary

Although targeted therapies and immune checkpoint inhibitors have improved patient outcomes, most metastatic renal cell carcinoma tumors eventually become resistant to treatment and progress. In this study, we used a syngeneic mouse model of metastatic renal cell carcinoma to investigate how gene expression changes during disease progression and in response to treatment with cabozantinib, an anti-PD-1 antibody, or both. We found that different treatments activated distinct molecular pathways in metastatic tumors. These treatment-specific changes influenced how tumors responded to subsequent therapies. Our findings suggest that the biology of metastatic renal cell carcinoma evolves depending on prior treatment, and that selecting second-line therapies based on these molecular changes may improve outcomes. This research provides insights that could help guide future treatment strategies and support the development of more effective, personalized therapy sequences for patients with metastatic renal cell carcinoma.

## 1. Introduction

Renal cell carcinoma (RCC) is a common malignant disease, with 434,840 cases reported worldwide by 2022 [1]. Surgical resection is an essential treatment for localized or locally advanced disease, whereas unresectable disease is generally incurable. The introduction of molecular targeted therapy has improved the treatment outcomes and prognosis of metastatic RCC (mRCC) compared with the era of cytokine therapy. Vascular endothelial growth factor receptor-tyrosine kinase inhibitors (VEGFR-TKIs) have been the standard [2,3,4,5,6], and mammalian targets of rapamycin inhibitors (mTORi) have demonstrated clinical benefits [7,8]. Immune checkpoint inhibitors (ICI) have a significant impact on mRCC treatment. After its use as salvage therapy [9], ICI has become the core of combined immunotherapy, which is the current standard first-line treatment strategy. Combining anti-PD-1 and anti-CTLA-4 antibodies, nivolumab and ipilimumab, has demonstrated long-term survival benefits [10]. In addition, combinations of anti-PD-1 or PD-L1 antibodies with VEGFR-TKIs, such as pembrolizumab plus axitinib [11], avelumab plus axitinib [12], nivolumab plus cabozantinib [13], and pembrolizumab plus lenvatinib [14], have demonstrated efficacies comparable to those of ICI combination therapy. However, according to pivotal phase 3 clinical trials, the progression-free survival of each regimen is no longer than 2 years. While a few patients achieve long-term disease control through treatment, most patients require sequential salvage therapy. In this setting, belzutifan, a HIF-2α inhibitor, demonstrated promising results in phase 3 clinical trials [15]. However, an optimal sequential therapy has not yet been established.

The mechanisms underlying drug resistance in renal cell carcinoma (RCC) remain a topic of ongoing discussion. RCC is known for its intratumoral heterogeneity [16]. This, along with tumor progression and metastasis, suggests that drug sensitivity can vary between different tumor sites within the same individual. Furthermore, exposure to therapeutic agents may activate alternative molecular pathways that promote tumor progression. This is known as the “escape phenomenon” or a “bypass pathway” [17,18,19,20,21,22]. Moreover, exposure to therapeutic agents may induce changes in tumor biology, such as epithelial-to-mesenchymal transition (EMT), as well as alterations in the tumor microenvironment that affect the immune response [21,22].

The gold standard methods for gene expression analysis are microarrays and quantitative RT-PCR. However, with the evolution of next-generation sequencing technologies, RNA sequencing has been used for gene expression analysis [23]. RNA sequencing allows simultaneous analysis of multiple gene pathways. In this study, we aimed to clarify the gene expression alterations in mRCC induced by disease progression and exposure to treatments, including ICIs, using a mouse in vivo subclinical model. Furthermore, we verified the relationship between the gene expression analysis results and the efficacy of subsequent treatments.

## 2. Materials and Methods

### 2.1. Cells and Culture Conditions

The murine RCC cell line, RENCA, was purchased from the American Type Culture Collection (CRL-2947, Rockville, MD, USA). The cell line originates from renal tubule–derived adenocarcinoma in a BALB/c mouse, although it retains intact VHL protein [24]. Cells were cultured in RPMI-1640 medium supplemented with 10% fetal bovine serum), 100 IU/mL penicillin, and 100 µg/mL streptomycin. All cultures were maintained at 37 °C in a humidified incubator with 5% CO_2_.

### 2.2. Mouse Orthotopic Syngeneic Metastatic Renal Cell Carcinoma Model

Male BALB/c mice (6–8 weeks old, approximately 20 g in body weight) were purchased from Kitasato Animal Supply Co. (Sapporo, Hokkaido, Japan) and used for in vivo experiments. Under general anesthesia with isoflurane (1.5–2.0%) and oxygen at a flow rate of 2 L/min, RENCA cells (5 × 10^4^ cells suspended in 40 µL of PBS) were implanted under direct visualization into the subcapsular space of the left kidney using a 30-gauge needle attached to a 0.5 mL syringe.

Two weeks after implantation, contrast-enhanced microcomputed tomography (CT) was performed weekly to confirm the formation of primary renal tumors and the development of lung metastases. Tumor growth and lung metastasis were monitored longitudinally.

All animal experiments were approved by the Institutional Animal Care and Use Committee of Sapporo Medical University and performed in accordance with national and institutional regulations.

### 2.3. Treatment and Experiment Protocol

**Figure 1:**
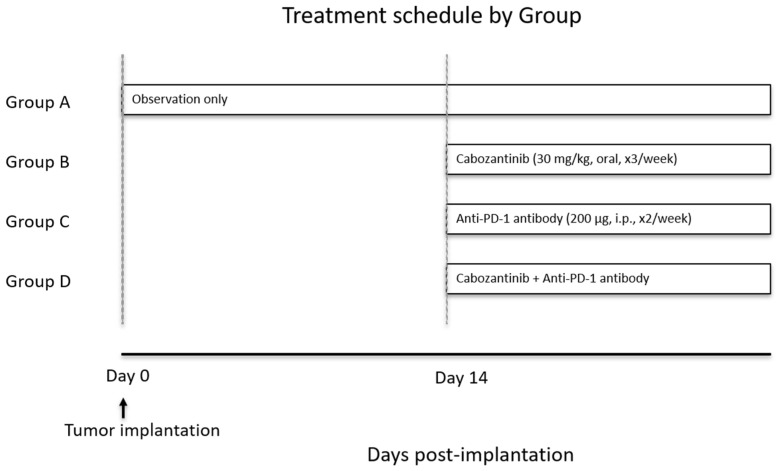
RENCA cells were implanted under the left renal capsule of BALB/c mice. Therapeutic interventions were initiated on day 14 post-inoculation. Group A received no treatment and was observed without intervention. Group B received cabozantinib monotherapy, Group C was treated with anti-PD-1 antibody monotherapy, and Group D received combination therapy with cabozantinib and anti-PD-1 antibody.

**Figure 2:**
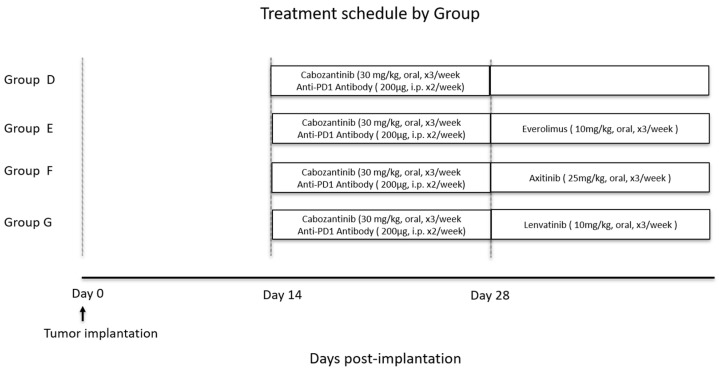
Groups E to G received combination therapy for 2 weeks, followed by a treatment switch to axitinib (Group E), everolimus (Group F), or lenvatinib (Group G), respectively.

All mice underwent weekly body weight measurements and were monitored daily for health status. After confirming tumor establishment using micro-CT, the mice were randomly assigned to first-line treatment groups (Figure 1). Group A was observed until cancer-related death without the administration of any therapeutic agent. Group B received cabozantinib at a dose of 30 mg/kg administered orally once daily for 3 days per week. Group C was treated with nivolumab at 200 μg, administered intraperitoneally once daily for 2 days per week. Group D received a combination therapy consisting of cabozantinib and nivolumab. Three additional groups were treated with sequential second-line therapy with everolimus, axitinib, and lenvatinib for apparently progressive tumor after combination therapy with cabozantinib and nivolumab (Figure 2). In all cases, a 100% or greater increase in tumor size was observed at the initiation of second-line treatment. These agents were selected based on their clinical relevance in the treatment of mRCC. Everolimus and lenvatinib were chosen for their distinct targets—mTOR and FGFR/VEGFR, respectively—compared to cabozantinib, which targets VEGFR, AXL, and MET [25]. Axitinib was included because it shares a common target with cabozantinib (VEGFR) but is more selective in its inhibition. In Group E, after two weeks of the combination therapy, everolimus was administered at 10 mg/kg orally three times per week from the third week onward. In Group F, after 2 weeks of combination therapy, axitinib was administered orally at 25 mg/kg three times per week from the third week onward. Similarly, in Group G, after two weeks of combination therapy, lenvatinib was administered orally at 10 mg/kg three times per week. The dosages per administration of each agent were determined with reference to previously published reports [26,27,28,29,30], and the frequency of oral administration was modified and standardized for the current study. Tumor-bearing mice were monitored daily, and if they exhibited a severe decline in activity, experienced weight loss exceeding 20% of their initial body weight, or showed labored breathing, they were humanely euthanized in accordance with the animal care guidelines of our facility (via cervical dislocation or a lethal dose administered under anesthesia). For survival analysis, both spontaneous death and euthanasia due to tumor burden were classified as “tumor-related death”.

### 2.4. Evaluation of Objective Response

All mice underwent contrast-enhanced in vivo micro-CT imaging in the second week after tumor implantation. Thereafter, micro-CT scans were performed weekly to assess the size of the primary renal tumor and the presence of distant metastases. A nonionic iodinated contrast agent approved for clinical use in humans was administered via tail vein injection immediately before each scan. Tumor volume in the kidney was calculated using the formula: longest diameter × shortest diameter × shortest diameter/2 and measured longitudinally over time.

### 2.5. Evaluation Criteria

The following endpoints were defined. In the untreated observation Group A (Group A), the consistency of tumor engraftment and the development of lung metastases were assessed to validate the reproducibility of the orthotopic metastatic RCC model. Using this established model, tumor growth dynamics and overall survival were analyzed in Group A and the first-line treatment groups (Groups B–D). Subsequently, RNA sequencing was performed on tumor tissues extracted from the kidney and lung metastases of tumor-bearing mice in Group A to evaluate gene expression differences between the primary and metastatic sites. RNA sequencing was conducted on lung metastasis samples collected from Groups B–D to assess the treatment-induced transcriptional changes. In addition, immunohistochemical staining using various antibodies was performed to visualize treatment-related differences in protein expression. Finally, tumor growth and survival outcomes were evaluated in the sequential therapy groups (Groups D–G). Animals that died due to accidental causes or technical issues were excluded from the analysis.

### 2.6. RNA Sequencing

Total RNA was extracted from the tumor tissues and submitted to Macrogen Inc. (Seoul, Republic of Korea) for library preparation and RNA sequencing. Libraries were prepared using the TruSeq Stranded mRNA Sample Prep Kit (Illumina, San Diego, CA, USA), and sequencing was performed using the Illumina NovaSeq X Plus platform with paired-end 101 bp reads. The resulting FASTQ files were assessed for quality using FastQC (v0.11.7) [31]. Adapter sequences were removed, and low-quality bases were trimmed using Trimmomatic (v0.38) [32]. The cleaned reads were aligned to the mouse reference genome (mm10) using HISAT2 (v2.1.0) [33], and gene expression levels were quantified using StringTie (v2.1.3b) [34]. Transcript abundance was calculated as transcripts per million. Differentially expressed genes (DEGs) were identified using the edgeR package in the R/Bioconductor environment [35]. Genes with a fold change ≥ 2 and *p*-value < 0.05 were considered significantly differentially expressed. Functional enrichment analysis based on Gene Ontology (GO) was conducted using g:Profiler [36].

### 2.7. Statistical Analysis

Statistical analyses were conducted using EZR software (version 1.40; Saitama Medical Center, Jichi Medical University, Japan). Statistical significance was set at *p* < 0.05. All results are expressed as the mean ± standard deviation. Comparisons between the two groups were performed using a two-sided Student’s *t*-test and Mann–Whitney U test. Mouse survival following treatment initiation was evaluated using the Kaplan–Meier method, and group differences were examined using the log-rank test.

## 3. Results

### 3.1. Establishment of Mice Orthotopic Metastatic Renal Cell Carcinoma Model

**Figure 3:**
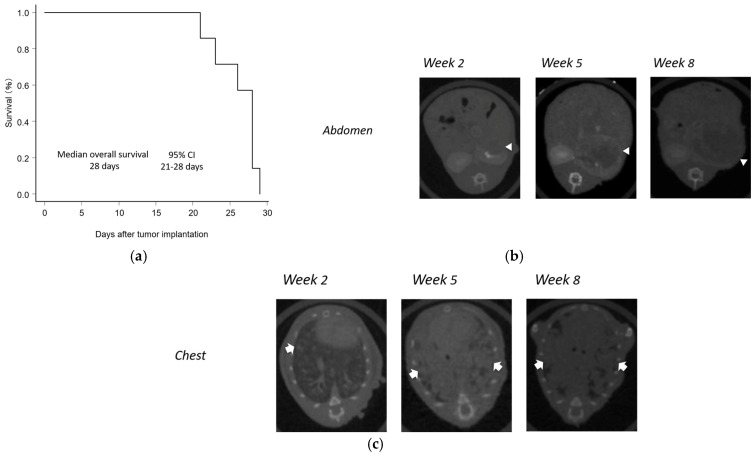
Establishment of mouse orthotopic syngeneic metastatic renal cell carcinoma model. (**a**) Overall survival. (**b**) Contrast-enhanced CT of the abdomen. RENCA cells were implanted under the left renal capsule. Two weeks after implantation, contrast-enhanced abdominal CT imaging was performed to confirm tumor engraftment. The left renal tumor progressively increased in size over time. (**c**) CT imaging of the chest in BALB/c mice over time. From the third week after tumor implantation, multiple lung metastases gradually appeared, with both the number and size of lesions increasing progressively.

Following the implantation of RENCA cells, all mice succumbed to cancer progression, with a median survival time of 28 days (95% CI, 21–28 days; Figure 3a). The autopsy revealed metastases to the abdominal lymph nodes, lungs, spleen, liver, abdominal wall, and intestine in 100%, 100%, 100%, 75.0%, 12.5%, and 12.5% of cases, respectively. In contrast, no tumors were observed in the contralateral kidney or bone. Figure 3b,c show the development of primary renal tumors and lung metastases as confirmed using micro-CT. By week 2, tumor engraftment was confirmed in all cases, and the primary renal tumors increased in size over time (Figure 3b). Lung metastases appeared from week 3 onward, with a subsequent increase in the number and size of the metastatic lesions (Figure 3c). These trends were consistently observed in Group A, indicating that this model reliably recapitulated an orthotopic metastatic renal cell carcinoma model. Accordingly, in the first-line treatment groups (Groups B–G), the treatments were started on day 14 after cancer cell implantation. The renal tumor was measurable, whereas the size of the lung metastasis could not be evaluated on micro-CT because of accompanying lymphangitic carcinomatosis and pleural effusion.

### 3.2. RNA Sequencing in the Kidney Tumor and Lung Metastasis

**Figure 4:**
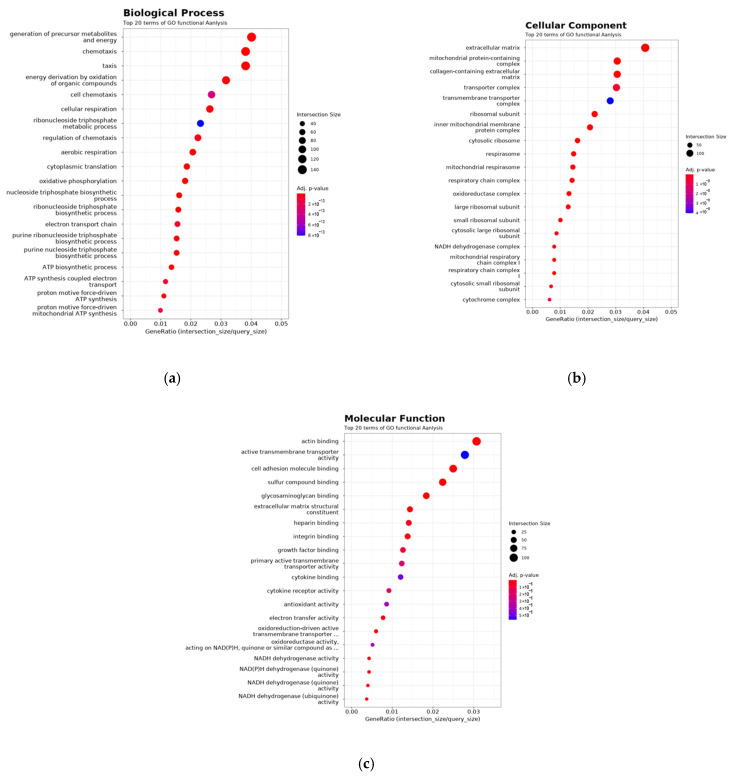
RNA was extracted from lung metastatic lesions and primary renal tumors (left kidney) in an orthotopic renal tumor model, in which RENCA cells were implanted under the left renal capsule of BALB/c mice and monitored without treatment. RNA sequencing was performed, and differential gene expression analysis was conducted to compare the transcriptomic profiles between the metastatic and primary tumor sites. Gene Ontology (GO) enrichment analysis was applied to the differentially expressed genes. (**a**) Biological Process, (**b**) Cellular Component, and (**c**) Molecular Function show the top 20 enriched GO terms in each category.

RNA sequencing was performed on primary kidney tumors and the corresponding lung metastases obtained from the observation group. DEGs between primary renal tumors and metastatic lung lesions were subjected to GO enrichment analysis. In the biological process category, the genes upregulated in lung metastases were enriched in pathways related to chemotaxis, energy production via organic compound oxidation, cellular respiration, and oxidative phosphorylation (Figure 4a). In the cellular component category, highly expressed genes were associated with structural components such as mitochondrial protein complexes, respiratory chain complexes, ribosomal subunits (small and large), extracellular matrix, and transporter complexes (Figure 4b). In the molecular function category, genes involved in actin binding, cell adhesion molecule binding, growth factor binding, electron transfer activity, and oxidoreductase activity were upregulated during lung metastasis (Figure 4c).

**Figure 5:**
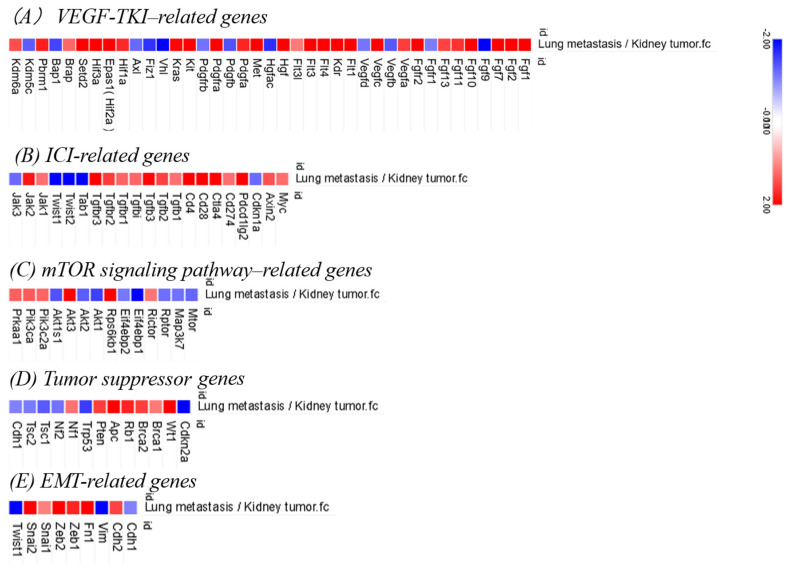
Heatmap comparing gene expression between lung metastases and primary kidney tumors in Group A (observation only). In the orthotopic renal tumor model, RNA was extracted from lung metastases and primary renal tumors (left kidney) in BALB/c mice from Group A (observation only), and RNA sequencing was performed. Differential expression analysis based on fold change (Fc) was conducted to compare the gene expression profiles between the two tissues. The heatmap shows expression patterns for selected gene sets: (**A**) VEGF-related genes, (**B**) immune checkpoint inhibitor (ICI)-related genes, (**C**) mTOR signaling pathway-related genes, (**D**) tumor suppressor genes, and (**E**) epithelial–mesenchymal transition (EMT)-related genes. Gene expression levels are represented as Z-scores, with red indicating higher expression and blue indicating lower expression.

With regard to VEGF-TKI-related genes, the upregulation of lung metastases was observed for Fgf2, Fgf7, Fgf13, Vegfc, Flt1, and Kdr (Figure 5A). Among ICI-related genes, increased expression was noted for Pdcd1lg2 (PD-L2), Ctla4, and Tgfbr3 (Figure 5B). In the mTOR signaling pathway, Rps6kb1 and Akt3 were upregulated (Figure 5C). Partial upregulation of tumor suppressor genes was observed in Apc, Wt1, and Rb1 cells (Figure 5D). Among the EMT-related genes, elevated expression of Zeb1, Zeb2, Snai2, and Fn1 and downregulation of Cdh1 were observed (Figure 5E).

### 3.3. Objective Response and Overall Survival in Each First-Line Treatment

**Figure 6:**
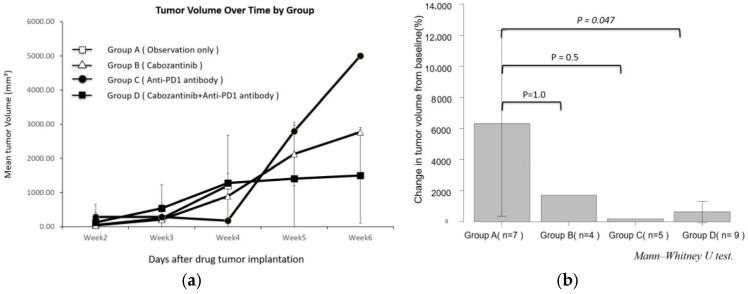
(**a**) Time course of mean tumor volume in Groups A–D, measured using contrast-enhanced abdominal micro-CT following tumor implantation in the left kidney. Data are expressed as the mean ± standard deviation (SD). For timepoints where only a single animal was evaluable (n = 1), the individual value is shown without error bars. (**b**) Percentage change in tumor volume from baseline to week 4 was compared among Groups A–D. Statistical analysis was performed using the Mann–Whitney U test, and differences were considered significant at *p* < 0.05.

Changes in the diameter of the primary renal tumor over time in response to treatment interventions are shown in Figure 6. Although tumor growth accelerated after week 4 post-implantation in all groups, Group D demonstrated a trend toward attenuated primary tumor growth relative to the other groups (Figure 6b).

**Figure 7:**
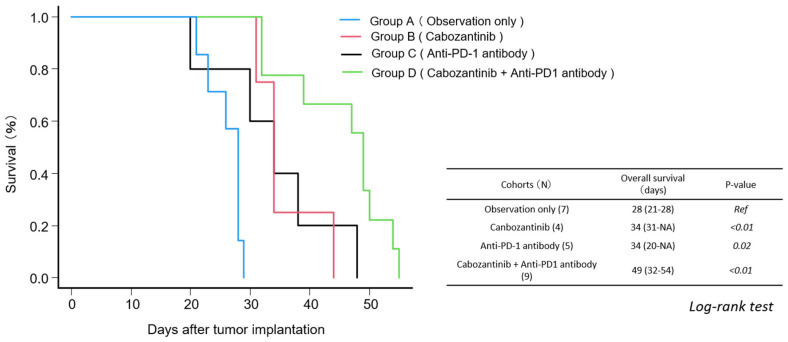
Kaplan–Meier survival curves for each treatment group. Statistical analysis of survival duration was performed using the log-rank test. A *p* value of <0.05 was considered statistically significant.

Survival outcomes for Groups A–D are shown in Figure 7. Groups B–D, which received first-line anticancer treatment, demonstrated significantly prolonged survival compared to Group A. However, all mice in these treatment groups ultimately succumbed to cancer progression, suggesting the emergence of treatment-resistant diseases in each group. At autopsy, the distribution and burden of metastatic sites did not differ between the treatment groups.

### 3.4. RNA Sequencing After First-Line Treatment

**Figure 8:**
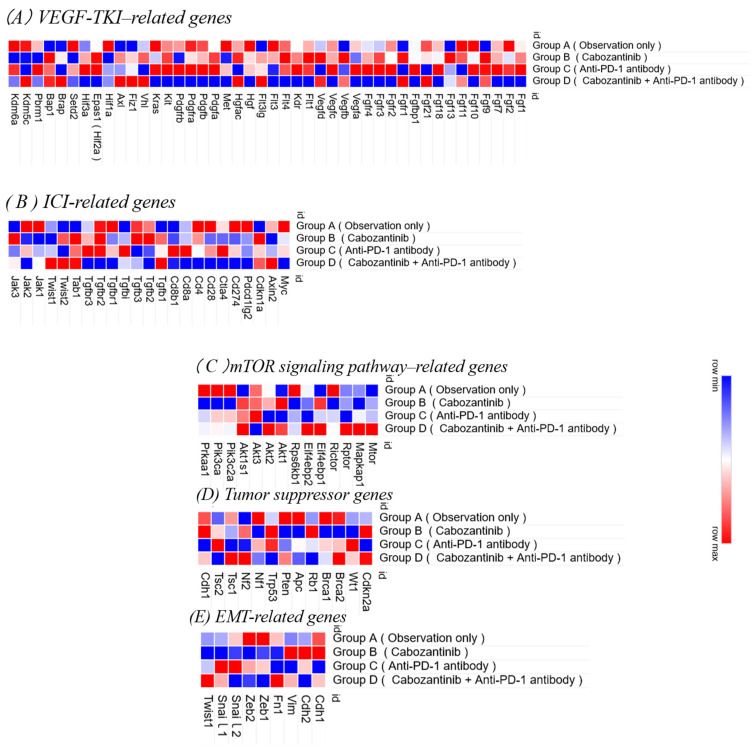
Heatmaps of RNA sequencing data from lung metastases in each treatment group. RNA was extracted from lung metastatic lesions in the RENCA tumor model and subjected to RNA sequencing. The heatmap shows expression patterns for selected gene sets: (**A**) VEGF-related genes, (**B**) immune checkpoint inhibitor (ICI)-related genes, (**C**) mTOR signaling pathway-related genes, (**D**) tumor suppressor genes, and (**E**) epithelial–mesenchymal transition (EMT)-related genes. Gene expression levels are represented as Z-scores, with red indicating higher expression and blue indicating lower expression.

RNA sequencing analysis of lung metastases from Groups A–D revealed distinct gene expression changes associated with treatment interventions (Figure 8). Activation of the VEGF/VEGFR and HGF/MET pathways was observed in the cabozantinib monotherapy group, whereas these pathways were downregulated in the combination therapy group. The FGF/FGFR pathway was upregulated in monotherapy groups, with a similar trend observed in part of the combination group. HIF-2α expression was elevated in the monotherapy groups and suppressed in the combination group (Figure 8A). In the anti-PD-1 antibody monotherapy group, increased expression of CTLA4 and CD8 was detected, whereas the combination therapy group exhibited downregulation of genes associated with lymphocyte activation. The combination group showed broad upregulation of genes involved in the mTOR signaling pathway (Figure 8C). Among the tumor suppressor-related genes, CDKN2a expression increased, whereas Trp53 expression decreased in the combination group (Figure 8D). In addition, the upregulation of TWIST and Fn1, which are associated with EMT, was observed (Figure 8E).

### 3.5. Objective Response and Overall Survival Following Second-Line Treatment After Anti-PD-1 Antibody + Cabozantinib Therapy

**Figure 9:**
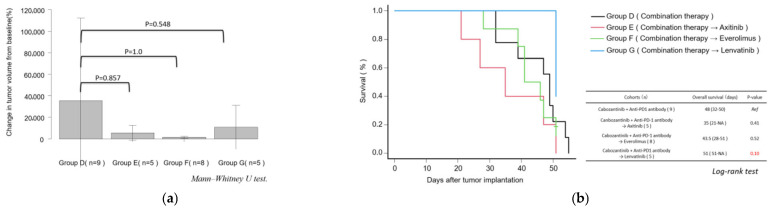
Results of second-line treatments. (**a**) Percentage change in tumor volume from baseline to week 6 was compared among Groups D–G. Statistical analysis was performed using the Mann–Whitney U test, and differences were considered significant at *p* < 0.05. (**b**) Kaplan–Meier survival curves for Groups D–G following second-line treatment. (A) Kaplan–Meier survival curves for each treatment group. (B) Statistical analysis of survival duration was performed using the log-rank test. A *p* value of <0.05 was considered statistically significant.

Subsequently, tumor growth and survival were evaluated in Group D, which received combination therapy with cabozantinib and anti-PD-1 antibody, and in Groups E–G, which received second-line treatment following combination therapy. The change in volume of the primary renal tumor is shown in Figure 9a. Although tumor growth appeared to be suppressed in Group F, the difference was not statistically significant. Similarly, the differences in overall survival between the groups were not statistically significant (Figure 9b).

### 3.6. RNA Sequencing After Second-Line Therapy

**Figure 10:**
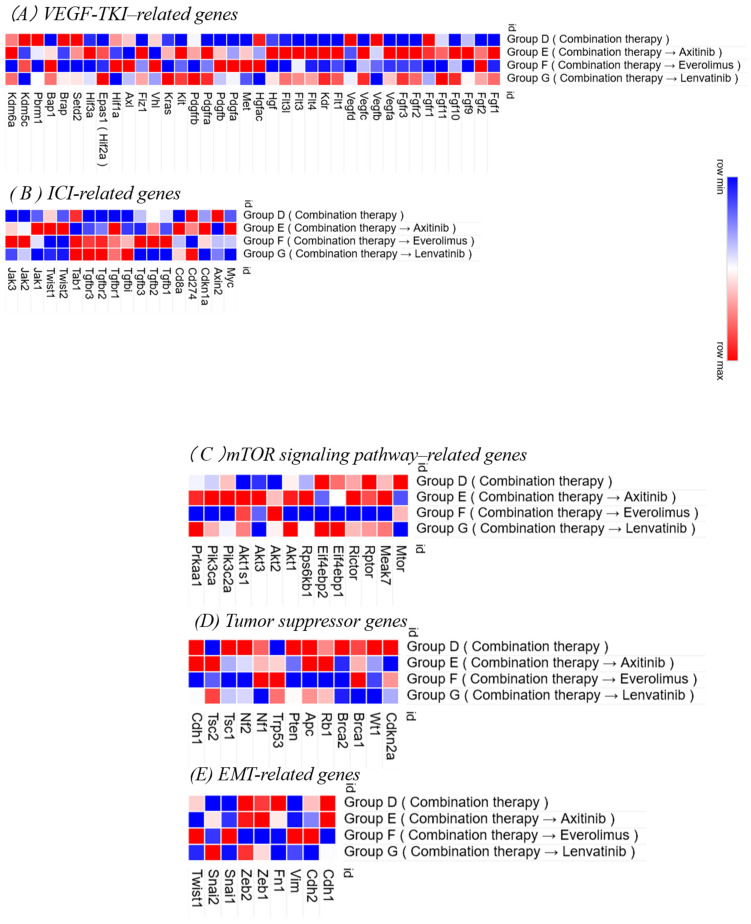
Heatmaps of RNA sequencing data from lung metastases in each second-line treatment group. RNA was extracted from lung metastatic lesions in the RENCA tumor model and subjected to RNA sequencing. The heatmap shows expression patterns for selected gene sets: (**A**) VEGF-related genes, (**B**) immune checkpoint inhibitor (ICI)-related genes, (**C**) mTOR signaling pathway-related genes, (**D**) tumor suppressor genes, and (**E**) epithelial–mesenchymal transition (EMT)-related genes. Gene expression levels are represented as Z-scores, with red indicating higher expression and blue indicating lower expression.

RNA sequencing analysis of lung metastases from Group D (treated with cabozantinib and nivolumab) and Groups E and G (treated with second-line therapies) revealed differential gene expression profiles (Figure 10).

Compared to Group D, Groups E (axitinib) and G (lenvatinib), in which the treatment was switched after 2 weeks, exhibited increased expression of VEGF-related genes, such as Vegfa, Vegfb, Flt1, and Kdr, as well as FGF pathway genes, including Fgf1, Fgf2, and Fgfr1–3. In contrast, Group F (everolimus), which received everolimus as a second-line agent, showed an upregulation of Hgf, Met, Pdgfa, Pdgfb, Pdgfra, and Pdgfrb, indicating enhanced activity of the HGF/MET and PDGF pathways. Furthermore, Groups G and F exhibited upregulation of TGF-β-associated genes relative to the other groups.

## 4. Discussion

Since the development of VEGFR-TKIs, no effective treatment has existed for mRCC for a long time [37]. VEGF is essential for angiogenesis to form the tissue microenvironment required for cancer cell survival. Additionally, VEGF directly affects cancer cells by promoting their proliferation and aggressiveness [38]. Therefore, VEGFR-TKIs act against endothelial cells and cancer cells themselves. Furthermore, the introduction of ICI has drastically changed treatment strategies for RCC and other malignant diseases [39]. In addition, VEGF plays a role in the “cancer-immunity cycle”. VEGFR-TKIs may remodel the tumor microenvironment to enhance cancer immunogenicity [40]. This suggests that combining VEGF-TKIs with ICIs may have a synergistic effect. Recent clinical trials have shown that these combination therapies provide better clinical outcomes than sequential monotherapies of each agent [11,12,13,14]. These findings support the synergistic effect of combining VEGF-TKIs and ICI for mRCC. However, clinical trials have shown that the effectiveness of combination therapy with VEGF-TKI and ICI has limitations. The progression-free survival of each regimen is no longer than 2 years. Several mechanisms of treatment resistance have been proposed; however, they remain to be fully elucidated.

Controlling metastatic disease is crucial for the treatment of advanced cancer. Although aggressive treatments have been attempted, advanced metastatic disease can develop resistance to treatment [41]. Martínez-Jiménez et al. evaluated the whole-genome sequencing data of unpaired primary and metastatic solid tumor cohorts and showed that RCC has a large genomic discrepancy between primary and metastatic diseases [42]. Gulati et al. performed whole-exome and whole-transcriptome sequencing of unpaired primary and mRCC specimens and revealed significant heterogeneity between primary tumors and metastatic sites [43]. Disease progression, the tumor microenvironment, or exposure to anticancer therapy may induce genomic alterations, leading to treatment resistance. In our study, RNA sequencing of the same individual showed a discrepancy in gene expression between the primary tumor and lung metastases without any treatment. This finding suggests that the biological characteristics of the disease can vary by metastatic site. These differences may exist in both cancer cells and the tumor microenvironment, even without treatment influence. In lung metastases, in addition to the majority of EMT-related genes shown in Figure 5E, TGF-β and CDKN2A were upregulated compared to the primary tumor. Both TGF-β and CDKN2A are key regulators of epithelial-to-mesenchymal transition (EMT) [44,45]. This molecular divergence may reflect the aggressive characteristics of metastatic disease, including enhanced migration, invasion, and extravasation, and may contribute to treatment resistance and immune evasion. A fundamental discordance exists in the sensitivity to anticancer treatment between primary tumors and metastatic disease.

RNA sequencing of lung metastases that progressed after first-line treatment showed a specific pattern. Genes associated with the targets of the first-line therapy were downregulated. Instead, upregulation of genes associated with other molecular pathways was observed. These results suggest that tumors survive and proliferate via the activation of compensatory molecular pathways under conditions of suppression of a pathway associated with a therapeutic target. This so-called “escape phenomenon” has been proposed by several in vitro studies as a mechanism of resistance to anticancer drugs, including tyrosine kinase inhibitors [17,18,19,20,21,22]. This phenomenon was suggested in an in vivo mRCC model for the first time. Multi-target agents like cabozantinib, which inhibit MET and AXL in addition to VEGFR signaling, are considered promising for overcoming drug resistance [46]. However, this study showed that cabozantinib eventually led to the upregulation of alternative signaling pathways. Additionally, the effects of ICI therapy were evaluated. The gene expression profile after combination therapy with ICI and VEGF-TKIs was specific and differed from that after monotherapy. This finding reflects the synergistic effects of this combination, which may influence additional molecular targets. Furthermore, adequate sequential treatment after ICI + VEGFR-TKI combination therapy may be specific and different from that after VEGFR-TKI or ICI monotherapy. In this study, RNA sequencing revealed upregulation of FGFR2 and genes associated with the mTOR signaling pathway in progressive tumors following combination therapy with an anti-PD-1 antibody and cabozantinib. In the sequential therapy setting, lenvatinib and everolimus were employed to target FGFR2 and mTOR, respectively. Their efficacy was compared with axitinib, a highly selective VEGFR inhibitor. However, lenvatinib and everolimus did not demonstrate superior efficacy as second-line treatments compared to axitinib. RNA sequencing showed that treatment with lenvatinib, after cabozantinib plus nivolumab, activated the VEGF and FGF pathways. These are the intended targets of lenvatinib. This suggests that treatment failure was not due to an escape mechanism. The possibility of insufficient treatment cannot be ruled out, and more intensive administration of the agent might help suppress tumor progression. Alternatively, other resistance mechanisms, such as alterations in drug metabolism, may contribute to treatment resistance [21,22]. In contrast, sequential treatment with everolimus led to reactivation of the HGF/MET and PDGF pathways. These pathways may have been suppressed by prior cabozantinib therapy. Meanwhile, the mTOR pathway was downregulated. Additionally, upregulation of TGF-β-associated genes was observed, suggesting the induction of immunosuppressive mechanisms [47]. Although treatment resistance is multifactorial, these findings indicate that escape phenomena may represent a significant mechanism even during sequential therapy. In lung metastases, genes associated with the mTOR pathway, oxidative phosphorylation, and TGF-β signaling were upregulated. Furthermore, treatment with cabozantinib and nivolumab resulted in additional upregulation of mTOR and TGF-β. These pathways are known to play crucial roles in the metabolic plasticity of cancer stem cells [48], supporting the notion that adaptive metabolic reprogramming contributes to therapeutic resistance and tumor persistence.

This study has certain limitations. The most common subtype of RCC is clear cell RCC, which is associated with the inactivation of the VHL protein via genetic or epigenetic mechanisms. Although the RENCA model is widely used in murine RCC studies, it does not fully replicate the molecular characteristics of human clear cell RCC, particularly due to the lack of VHL mutations [24]. This limitation may affect the translatability of certain findings, especially those involving the VHL/HIF signaling pathway, which plays a central role in the pathogenesis of human clear cell RCC. In this study, an immunocompetent model was used to evaluate the effects of ICI treatment. Moreover, genomic changes were not evaluated in this study, whereas transcriptome analysis was performed. Genetic changes may be induced during proliferation, metastasis, or exposure to anticancer treatments; however, this was not addressed in this study. Furthermore, bulk RNA sequencing cannot distinguish gene expression in cancer cells from that in other cell types, such as lymphocytes, endothelial cells, and fibroblasts. Further studies using single-cell analyses are required to address this issue. Regarding the evaluation of anti-tumor effects and survival analysis, the sample size was too small to achieve sufficient statistical power. Additionally, more intensive administration of therapeutic agents might have influenced the outcomes. Despite these limitations, this study may be the first to reveal alterations in gene expression in mRCC during disease progression and the “escape phenomenon” in response to anticancer treatments, including immunotherapy, within the same individual.

## 5. Conclusions

The findings from this syngeneic mRCC mouse model suggest that alterations in gene expression are induced during both metastatic progression and in response to treatment. Combination therapy with a VEGFR-TKI and an immune checkpoint inhibitor triggered distinct molecular changes that were not observed with either monotherapy alone. These results highlight that the optimal target for sequential therapy in mRCC may vary depending on prior treatment.

## Data Availability

The data presented in this study are available. Further inquiries can be directed to the corresponding authors. The RNA sequencing dataset supporting the findings of this study is submitted to the NCBI Gene Expression Omnibus (GEO). The accession number is GSE302275.

## Abbreviations

The following abbreviations are used in this manuscript:

mRCC	metastatic renal cell carcinoma
RCC	renal cell carcinoma
VEGFR-TKIs	vascular endothelial growth factor receptor-tyrosine kinase inhibitors
mTORi	mammalian targets of rapamycin inhibitors
ICI	immune checkpoint inhibitors
EMT	epithelial-to-mesenchymal transition
CT	microcomputed tomography
DEGs	differentially expressed genes
GO	Gene Ontology
